# Retraction: Solar-Aligned Pictographs at the Paleoindian Site of Painel do Pilão along the Lower Amazon River at Monte Alegre, Brazil

**DOI:** 10.1371/journal.pone.0215119

**Published:** 2019-04-04

**Authors:** The PLOS ONE Editors

Following publication, the author informed the journal office that there are errors in the data reported in the article [1]. Specifically, the compass measurements used in Figs 9, 11, 12, and 13 are inaccurate due to magnetic distortion in the study area. When the compass measurements are corrected, the following results are no longer supported:

- The red alcove circle in Fig 13 faces 300°, not 270° and therefore it does not mark a spot where the sun would shine through the crevice directly beneath the circle on the day of the equinox. Instead, the circle may mark a position where sun would shine through the crevice at sunrise on the day of the winter solstice at ~114 ° (compass readings of 113°, 109°, 121° on different devices, averaged to 114.3°).
- The six vertical lines do not face 114°, and therefore do not mark the position of sunrise on the winter solstice.

The *PLOS ONE* Editors have considered the amendments to the data and have consulted with an external peer reviewer and a member of the Editorial Board. Following evaluation, there are concerns about the reliability of the results and conclusions, and some results are no longer supported, as follows:

- Compass measurements require independent validation; concerns remain regarding the accuracy of the data.
- Original key conclusions related to the intention behind the placement of the paintings are not fully supported.
- An astronomical alignment with sunrise on the true equinox was reported for the original compass measurements, and it is unclear what steps were taken to avoid a potential bias in the interpretation of the new measurements towards possible alternative astronomical alignments or explanations.

In light of the concerns raised about the accuracy of the data and the strength of support for the original conclusions, the *PLOS ONE* Editors retract this article.

CSD did not agree with retraction.

## References

[1] Davis CS (2016) Solar-Aligned Pictographs at the Paleoindian Site of Painel do Pilão along the Lower Amazon River at Monte Alegre, Brazil. PLoS ONE 11(12): e0167692 10.1371/journal.pone.0167692 27997553 PMC5173343

